# The Rhodium and Hafnium Co-Doping of Aluminide Coatings on Nickel and Superalloy

**DOI:** 10.3390/ma19214456

**Published:** 2026-10-23

**Authors:** Maryana Zagula-Yavorska

**Affiliations:** Faculty of Mechanical Engineering and Aeronautics, Rzeszow University of Technology, al. Powstańców Warszawy 12, 35-959 Rzeszów, Poland; yavorska@prz.edu.pl

**Keywords:** rhodium, hafnium, intermetallic coatings, oxidation resistance

## Abstract

Rhodium- and hafnium-co-doped aluminide coatings were manufactured via rhodium plating and further hafnizing combined with aluminizing on nickel and the CMSX-4 substrate using the Chemical Vapor Deposition CVD technique. Both co-doped coatings comprised two layers: an additive layer and an interdiffusion one. The additive layer contained the rhodium- and hafnium-doped β-NiAl phase. The interdiffusion layer on nickel contained the hafnium-doped γ’-Ni_3_Al phase, whereas that on the superalloy substrate contained the rhodium-doped β-NiAl phase. The chain of the hafnium-rich small particles was formed near the additive/interdiffusion layer border on both substrates. Rhodium- and hafnium-co-doped intermetallic coatings showed improved oxidation resistance compared to those doped only with rhodium. Moreover, rhodium and hafnium co-doping is more efficient than palladium and hafnium co-doping in reducing the oxidation rate of the aluminide compound on the CMSX-4 substrate.

## 1. Introduction

Gas turbines, such as jet engines, are widely used to power passenger and military aircraft, thanks to their reliability, safety, and high power [1]. Incoming air is compressed in the compressor and then flows to the combustion chamber, where fuel is supplied and the fuel–air mixture is combusted [2]. Hot exhaust gases flow to the turbine blades and create propulsion of the gas turbine. High efficiency of gas turbines is required. The best method for increasing gas turbine efficiency is to raise the hot gas’s operating temperature. Gas turbines are extremely prone to oxidation during operation, resulting in blade degradation that may lead to accidents. Thus, materials that are used on turbine blades have to maintain good mechanical properties and resistance to oxidation at a high temperature [3]. Nickel-based superalloys fulfill these requirements [4,5,6,7]. To increase the working temperature of turbine blades, a processing method was developed from casting through directional solidification to single crystallization. Turbine blades made from single-crystal nickel-based superalloys exhibit good creep resistance and fatigue strength at elevated temperatures. The regular morphology and a high γ′-phase volume fraction (>60%), which is uniformly precipitated within the γ-phase matrix, are reasons for the high mechanical properties of single-crystal nickel-based superalloys.

The thermal protection of single-crystal superalloys provides thermal barrier coatings (TBCs) [8]. The presence of internal cooling channels and thermal barrier coatings provides a reduction of temperature of up to 300 °C of single-crystal superalloy turbine blades [9]. Therefore, TBC application on the surface of the single-crystal superalloy turbine blades improves the performance of aircraft engines [10]. A multi-layered structure of TBCs ensures the reduction of thermal stresses within the coating. The ZrO_2_-stabilized Y_2_O_3_ is the top layer of the TBC system, while MCrAlY or aluminide (B2 crystal structure NiAl phase) is a bonding layer of the TBC system. NiAl has a relatively low density (about 6 g/cm^3^), a high melting point (1638 °C), and a high elastic modulus (240 GPa) [8]. These characteristics make NiAl the preferred bonding layer in the TBC system.

An aluminide coating comprises two distinct layers, which are the inter-diffusion zone (NiAl phase matrix with carbides or topologically close-packed precipitates) and the additive layer (NiAl phase) [11]. Topologically close-packed precipitates with a globular, needle-like, or plate-like morphology are enriched with tungsten, chromium, molybdenum, tantalum, or rhenium refractory substrate elements. A dense α-Al_2_O_3_ layer is formed on the surface of NiAl compounds during exposure to air at a temperature > 1000 °C [12]. Long exposure of NiAl coatings in harsh environments causes void formation between the oxide layer and the aluminide compound interface with subsequent oxide spallation [13]. Then, an intensive oxidation attack occurs at the spalled regions, shortening the service life of the coatings. Literature data indicate that hafnium [14], chromium [15], silicon [16], and platinum [17] doped into aluminide coatings slow the oxidation rate of coated superalloys. Platinum is the most effective element in slowing the oxidation rate of coated superalloys. Platinum, among other elements, reduces the coating degradation rate and oxide growth, mitigates scale spallation, and promotes α-Al_2_O_3_ formation. It was found that reactive elements, for example, Zr, Dy, or Hf, could improve the resistance to oxidation of the NiAl alloy at an elevated temperature [18]. Among all reactive elements, hafnium most improves the adherence of the oxide scale and reduces the rate of oxidation of superalloys. Hafnium has a high melting temperature (2225 °C) and high chemical reactivity with nitrogen, oxygen, carbon, and hydrogen [19]. Hafnium addition (0.1–1.0 wt.%) to an equiaxed nickel-based superalloy improved its mechanical properties at a high temperature [20]. A 0.5 at.% Hf doping substantially decreased cyclic oxidation of the aluminide coating [21]. The Hf-doped aluminide coatings were manufactured via the co-evaporation of Hf and NiAl using electron beam physical vapor deposition (EB-PVD) on a K3 nickel-base superalloy. The oxide layer formed after thermal exposure of the coating in air mainly comprised HfO_2_ and Al_2_O_3_. It was noticed that HfO_2_ kept good bonding with the underlying Al_2_O_3_ [21].

The state-of-the-art data indicate that mono-reactive element doping offers only limited benefits in increasing the NiAl alloy lifetime [22]. The strategy of co-doping to further increase the NiAl alloy lifetime has been used recently. Guo et al. [23] have shown that Y-La or Hf-Zr co-doping is more efficient in reducing the oxidation rate of the NiAl alloy compared to mono-doping. Unocic and Pint [24] highlighted that the scale adhesion of NiCrAl alloy Y and Hf co-doped was better than mono Y-doped.

Hf-doped (Ni,Pt)Al coatings were manufactured by firstly co-depositing a Pt-Hf electroplating and secondly aluminizing a single-crystal nickel-based superalloy [25]. An Hf-rich belt was identified between the interdiffusion zone and outer (Ni,Pt)Al zone of the coatings. The Hf-rich belt reduced the oxidation rate constant and decreased oxide layer rumpling of (Ni,Pt)Al coatings at 1100 °C in an air atmosphere. Moreover, hafnium postponed the transitional period of oxidation from unstable θ-Al_2_O_3_ to stable α-Al_2_O_3_ and delayed the β to γ’ phase transformation [25]. Zr-doped (Ni,Pt)Al coatings were produced by first co-depositing a Pt-Zr electroplating and secondly aluminizing a single-crystal nickel-base superalloy [26]. Zirconium doping reduced the oxidation rate constant and decreased oxide layer rumpling of (Ni,Pt)Al coatings at 1100 °C in an air atmosphere. Zirconium effectively postponed the θ-Al_2_O_3_-to-α-Al_2_O_3_ phase transformation and delayed β-to-γ’ phase degradation [26].

A combination of modificators provided a boosted coupling effect in improving the oxidation resistance of alloys. Nevertheless, the research considering two different modificators in intermetallic base coating is rare. Furthermore, the rhodium and hafnium co-doping of the coating is fully unknown. Therefore, the aim of the paper was rhodium and hafnium co-doping of an aluminide coating on a nickel and nickel-based superalloy. Moreover, the effect of rhodium and hafnium co-doping of aluminide coatings on the oxidation resistance of an Ni-based superalloy was assessed.

## 2. Materials and Methods

Rhodium layers on pure nickel and superalloy substrates were produced. CMSX-4, a single-crystal nickel-based superalloy of the second generation, was used as a substrate; the nominal chemical composition is listed in Table 1. Bal. (balance)—indicates that nickel (Ni) makes up the remainder of the alloy’s chemical composition after accounting for the other elements.

Substrates were ground and cleaned ultrasonically in ethanol (Alchem, Rzeszow, Poland), and subsequently, a 0.5 µm-thick rhodium layer was electrodeposited. The 0.1 g/dm^3^ Rh_2_(SO_4_)_3_ rhodium sulfate, 0.15 g/dm^3^ H_2_SO_4_ sulfuric acid, and a 0.010 g/dm^3^ H_2_SeO_4_ selenic acid bath were used during the electroplating process. Rhodium-electrodeposited specimens were aluminized using the chemical vapor deposition method in two ways. In the first way, the aluminizing of rhodium electrodeposited specimens was performed for 12h. The aluminum chloride vapor (AlCl_3_) was created in an external reactor 1 (IonBond, Company, Olten, Switzerland). The AlCl_3_ in the gaseous state was obtained at about 300 °C according to the following reaction (1):

6HCl + 2Al = 2AlCl_3_ + 3H_2_ (1)

Subsequently, aluminum chloride in the gaseous state in a stream of hydrogen was supplied to the reactor where superalloy specimens were situated. Aluminum chloride reacted with the main element of specimens—nickel; hence, the NiAl phase was formed according to the reaction (2):

2AlCl_3_ + 2Ni + 3H_2_ = 2NiAl + 6HCl (2)

In the second way, the aluminizing of rhodium-electrodeposited specimens consisted of five stages: (1) heating specimens from the ambient temperature to 1040 °C; (2) aluminizing for 1 h at 1040 °C; (3) aluminizing with hafnizing for 9 h at 1040 °C; (4) aluminizing for 1 h at 1040 °C; and (5) cooling specimens.

The hafnium chloride vapor (HfCl_4_) was created in an external reactor 2 (IonBond, Company, Olten, Switzerland).. The HfCl_4_ in the gaseous state was obtained at about 400 °C according to the reaction (3):

4HCl + Hf = HfCl_4_ + 2H_2_ (3)

Then, hafnium chloride in the gaseous state in a stream of hydrogen was supplied to the reactor where nickel and superalloy specimens were situated. Aluminum chloride and hafnium chloride reacted with the main element of specimens—nickel; hence the NiAl(Hf) phase was formed.

An oxidation test of coated specimens was conducted at 1100 °C in a muffle furnace (Czylok, Jastrzębie-Zdrój, Poland) with airflow. The temperature variation within the furnace did not exceed ±8 °C. Before the oxidation test, specimens were weighed and measured. Weighing accuracy was 0.0001 g, and measurements were replicated. The time duration of one cycle of oxidation was 20 h. After each cycle of oxidation, oxidized samples were removed from the muffle furnace and cooled to room temperature in the air. Samples were then inspected and weighed. Then, the weight change as a time function was recorded. The as-deposited rhodium- and hafnium-doped aluminide coating phase composition was identified using an ARL X’TRA x-ray diffractometer (XTRa ARL, Waltham, MA, USA) equipped with a Cu lamp with a voltage of 45 kV. The surface morphology and cross-section of as-deposited coatings were characterized using the scanning electron microscope (Hitachi America, Ltd., Iowa City, IA, USA) fitted with an energy dispersive X-ray spectroscope.

## 3. Results

### 3.1. Rhodium- and Hafnium-Doped Aluminide Coating on Nickel Substrate

**Figure 1:**
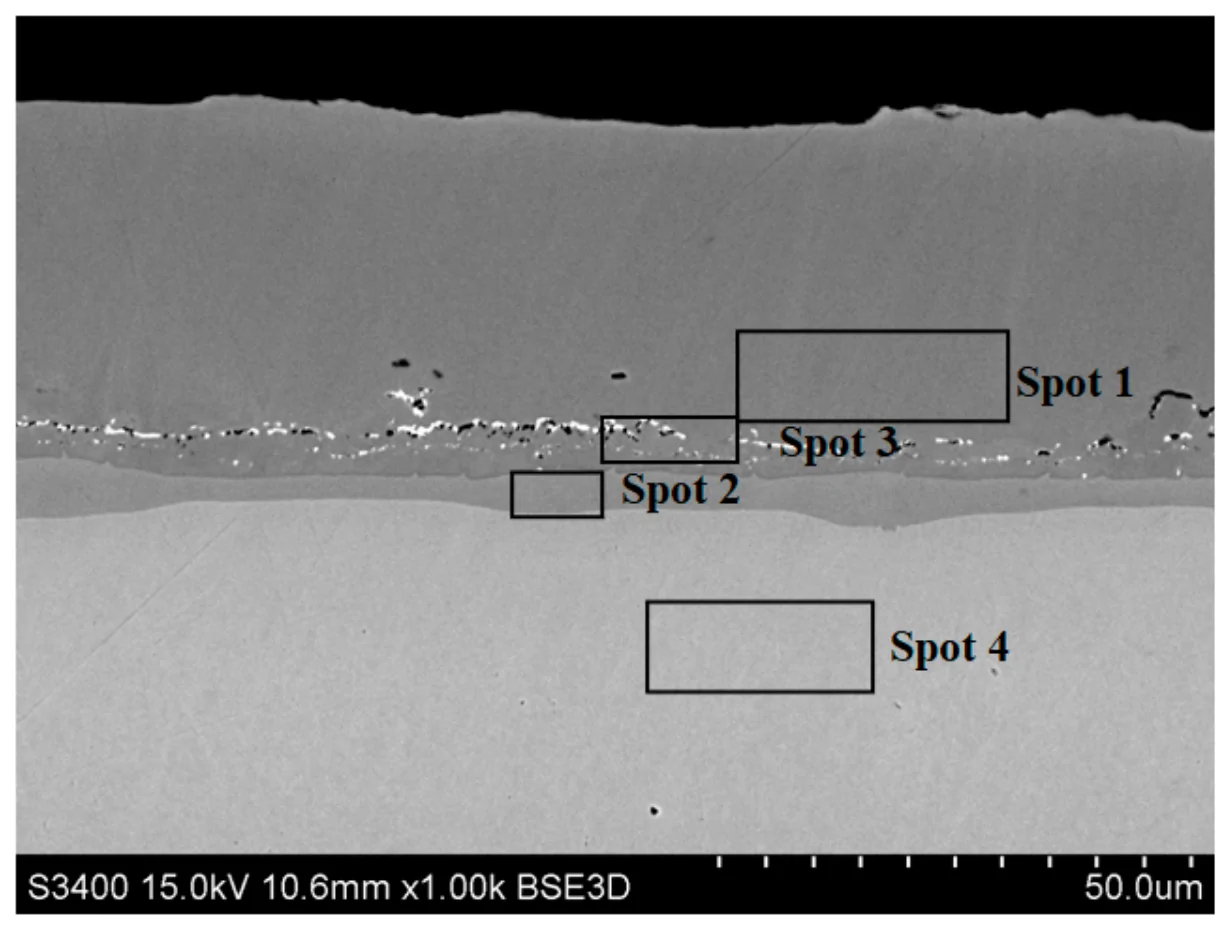
Cross-section microstructure of rhodium- and hafnium-doped aluminide coating on pure nickel.

**Figure 2:**
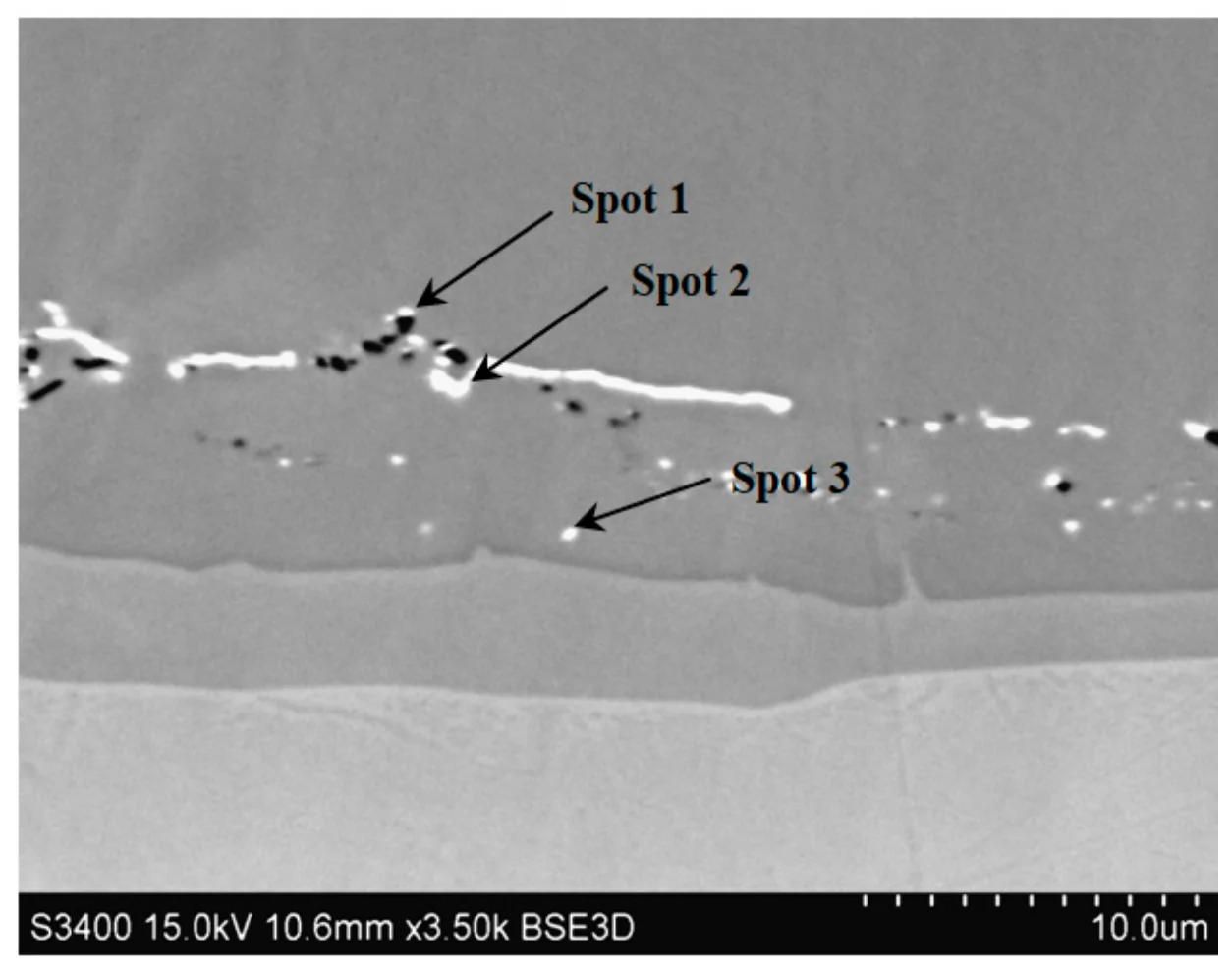
Cross-section of additive/interdiffusion layer border of rhodium- and hafnium-doped aluminide coating on pure nickel.

The rhodium- and hafnium-doped aluminide coating on a nickel substrate had a double-layer structure (Figure 1, Table 2). In the additive layer, on the top of the coating, the concentration of Al and Ni corresponded to the β-NiAl phase (Table 2, Spot 1). Additionally, small amounts of rhodium (0.5 at.%) and hafnium (0.3 at.%) were found. This indicated the formation of the hafnium- and rhodium-doped β-NiAl phase. The additive layer thickness was about 38 μm. The interdiffusion layer was located below the additive layer. The concentration of Al, Ni, and Hf in the interdiffusion zone corresponded to the hafnium-doped γ’-Ni_3_Al phase (Table 2, Spot 2). The interdiffusion layer thickness was about 4 μm. Kirkendall-like porosity with a hafnium-rich particle chain was observed near the additive/interdiffusion layer border (Figure 1 and Figure 2, Table 2, Point 3). Unequal flow of aluminum and nickel atoms was in the diffusion layer according to the Kirkendall theory. The value of the aluminum diffusion coefficient is lower than that of nickel (D_Al_ < D_Ni_). The unbalanced flux of aluminum and nickel atoms resulted in the differences in microvolume and caused stress in the diffusion zone. In the area of higher nickel content, the microvolume was reduced. As a result, vacancies were formed. Kirkendall porosity was generated when vacancies were coalesced [27]. The surface of the coating and additive/interdiffusion layer interface was rather flat.

Chain-like hafnium precipitates were distributed within pores near the additive/interdiffusion layer interface. Globular particles besides chain-like hafnium-rich precipitates were identified (Figure 2, Table 3).

**Figure 3:**
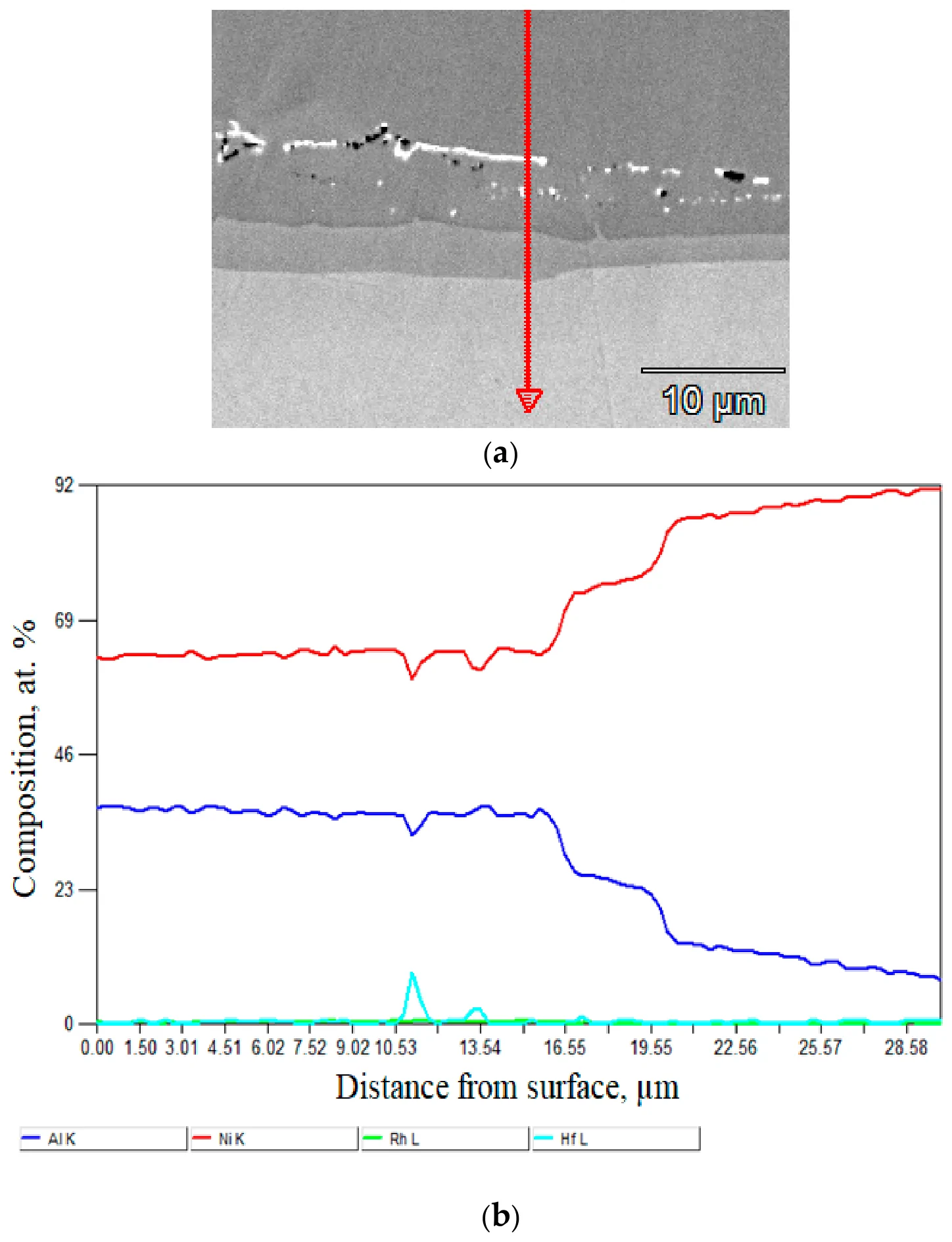
Microstructure (**a**) and composition based on the cross-section (**b**) of rhodium- and hafnium-doped aluminide coating on pure nickel.

The composition of the cross-section of rhodium- and hafnium-doped aluminide coatings is shown in Figure 3. The arrow indicates the location where the chemical composition was determined. The aluminum concentration increased from the substrate to the surface of the additive layer, while the nickel concentration was almost constant in the additive layer and increased in the interdiffusion layer. The results of hafnium content corresponded to bright, hafnium-rich precipitations near the additive/interdiffusion layer interface.

### 3.2. Rhodium- and Hafnium-Doped Aluminide Coating on CMSX-4 Substrate

**Figure 4:**
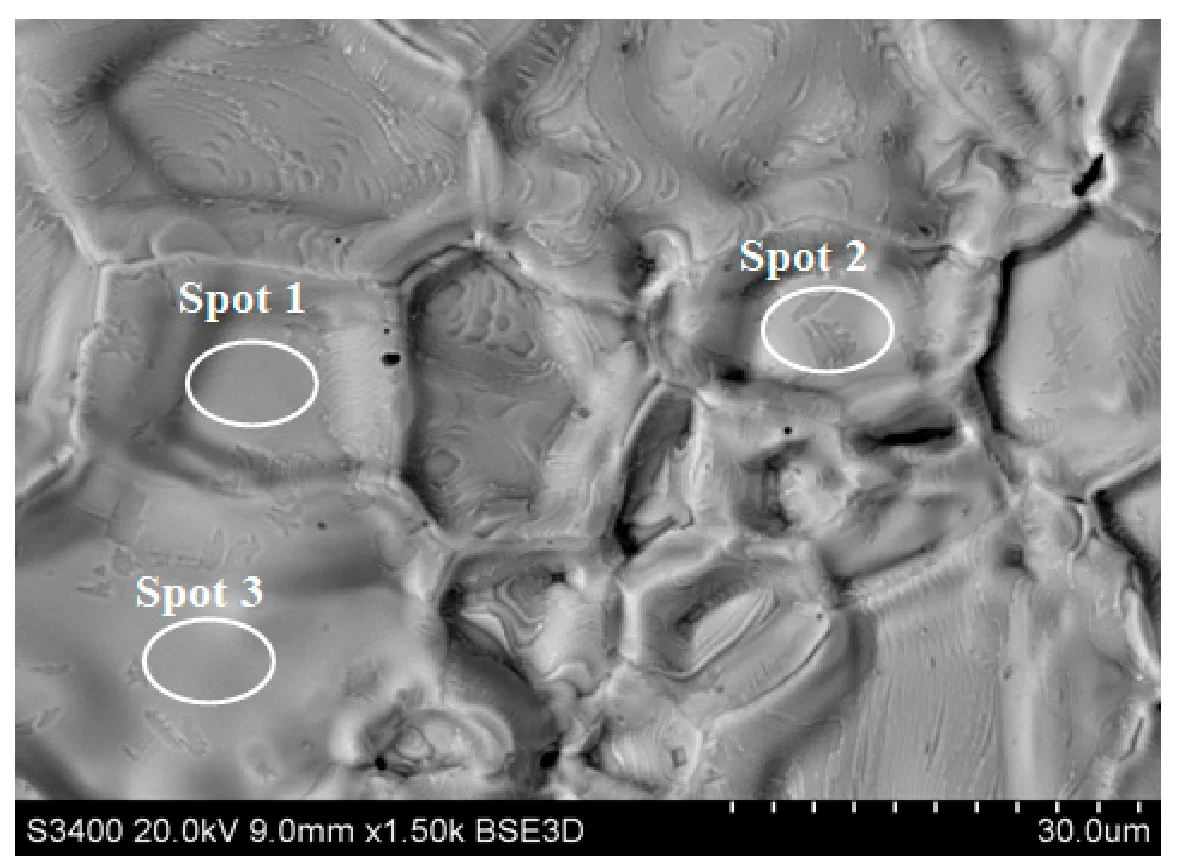
Surface morphology of rhodium- and hafnium-doped aluminide coating on CMSX-4.

The surface morphology of the rhodium- and hafnium-doped aluminide coating on the CMSX-4 substrate is shown in Figure 4. Grains divided by ridges of the grain boundary were observed. This morphology was typical for an aluminide coating obtained via aluminization with a gas phase [28,29,30]. The average size of grains of the coating surface was about 16 µm. The surface of the coating consisted mainly of aluminum and nickel with small concentrations of substrate elements, such as chromium and cobalt (Table 4). Aluminum and nickel contents suggested the β-NiAl phase presence (Table 4). Neither rhodium nor hafnium was identified on the surface of the rhodium- and hafnium-doped aluminide coating (Table 4).

**Figure 5:**
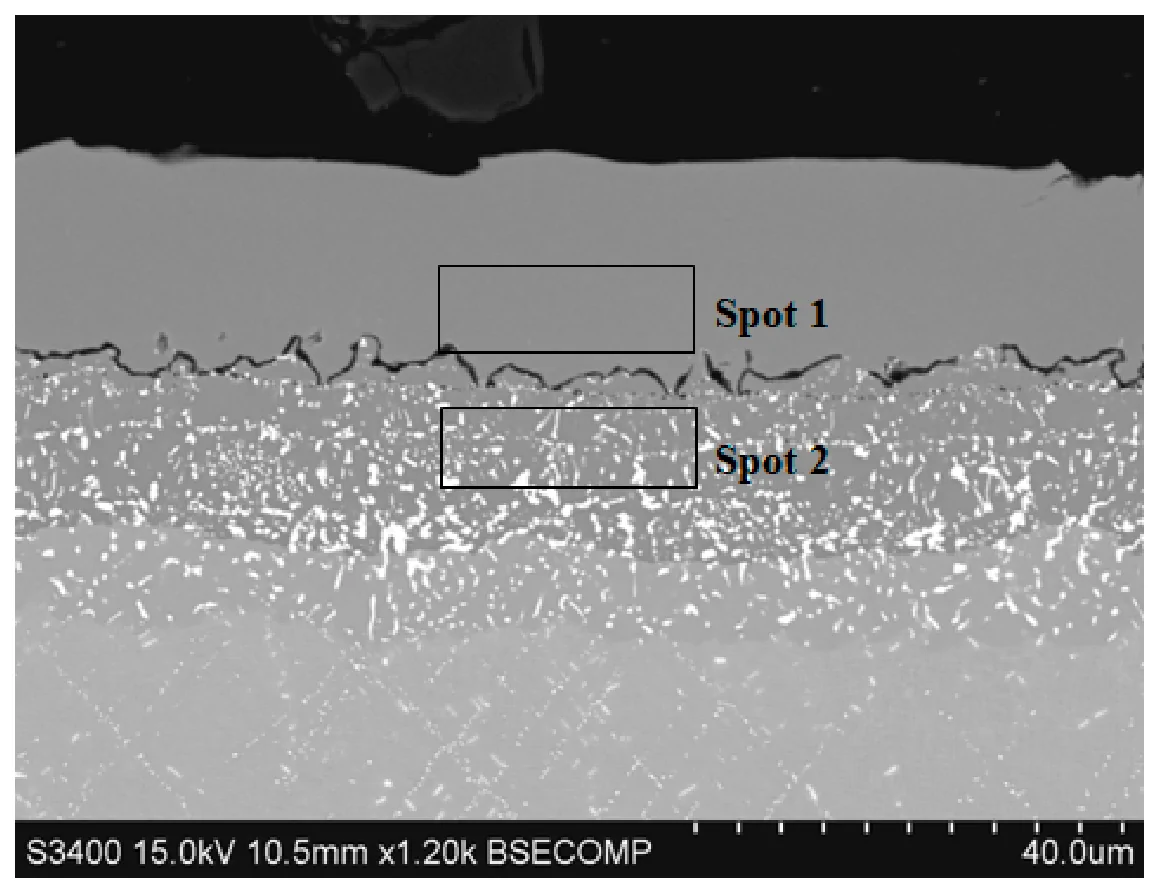
Cross-section microstructure of rhodium- and hafnium-doped aluminide coating on CMSX-4.

A rhodium- and hafnium-doped aluminide coating on the CMSX-4 superalloy comprised two layers: the additive, on the top of the coating, and the interdiffusion, below the outer one (Figure 5). The concentration of Al and Ni corresponded to the β-NiAl phase (Table 5, Point 1) in the additive layer. Rhodium (0.1 at.%) and hafnium (0.2 at.%) were also detected. Such results suggested a rhodium- and hafnium-doped β-NiAl phase. The additive layer thickness was about 20 μm. The surface of the coating was rather flat. The interdiffusion zone contained phases of refractory substrate elements (titanium, chromium, or rhenium) called topologically close packed (TCP) [31,32,33,34,35]. The interdiffusion layer thickness was about 16 µm. Kirkendall pores (dark areas) at the interface between the rhodium- and hafnium-doped β-NiAl additive layer and the interdiffusion layer were situated.

**Figure 6:**
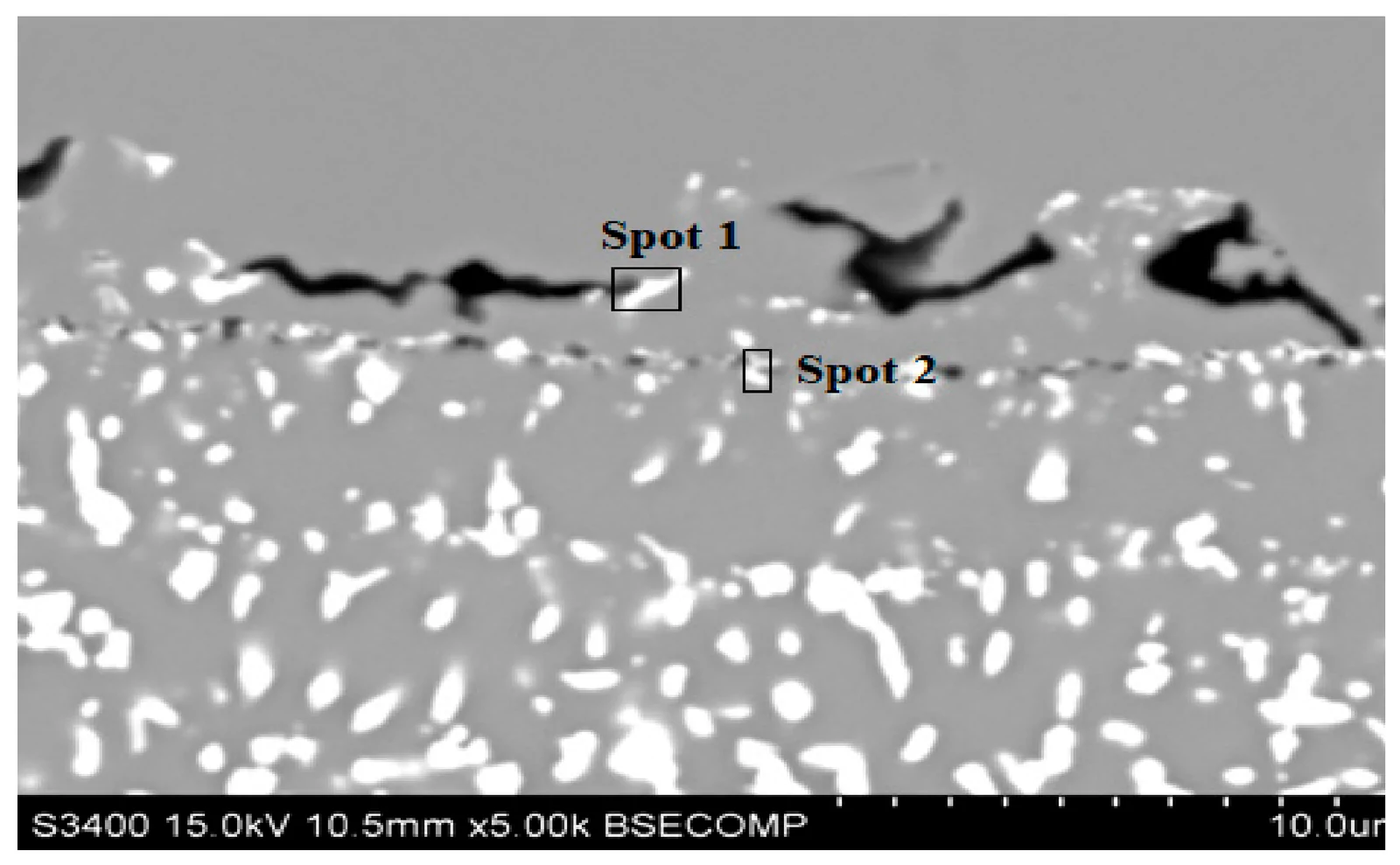
Cross-section of additive/interdiffusion layer border of rhodium- and hafnium-doped aluminide coating on CMSX-4.

**Figure 7:**
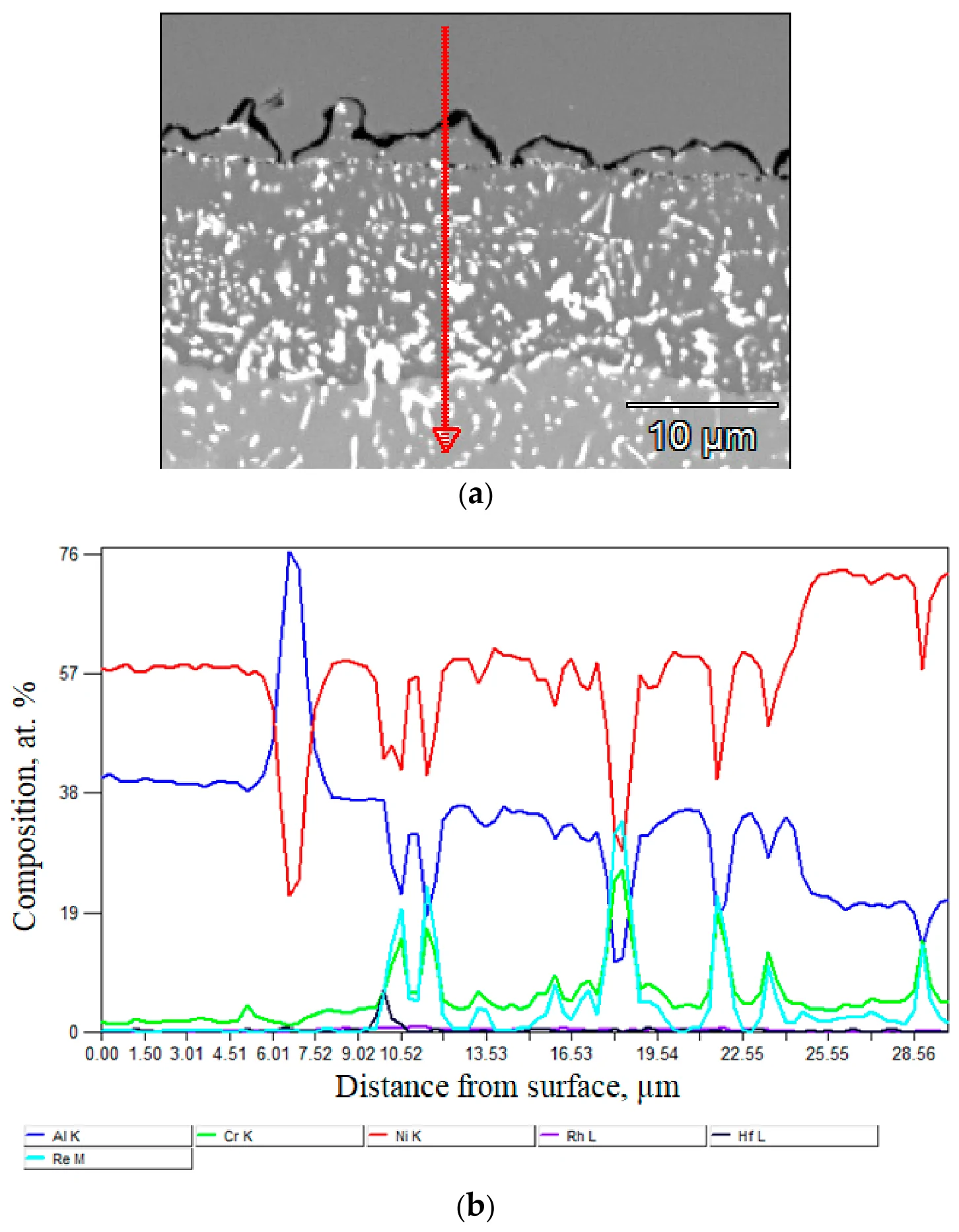
Microstructure (**a**) and composition on the cross-section (**b**) of rhodium- and hafnium-doped aluminide coating on CMSX-4.

Similar to the above-mentioned pure nickel substrate, the hafnium peak area in the element profile corresponded to the location of the bright-colored hafnium-rich particles. Hafnium-rich precipitates were distributed near the additive/interdiffusion layer interface (Figure 6 and Figure 7, Table 6). The Energy Dispersive Spectroscope EDS spot analysis suffers from the electron-beam interaction–volume effect, which inevitably collects mixed signals from both the precipitate and surrounding matrix. Therefore, Spot 2 in Table 6 represents the composition of the precipitate and the adjacent matrix.

**Figure 8:**
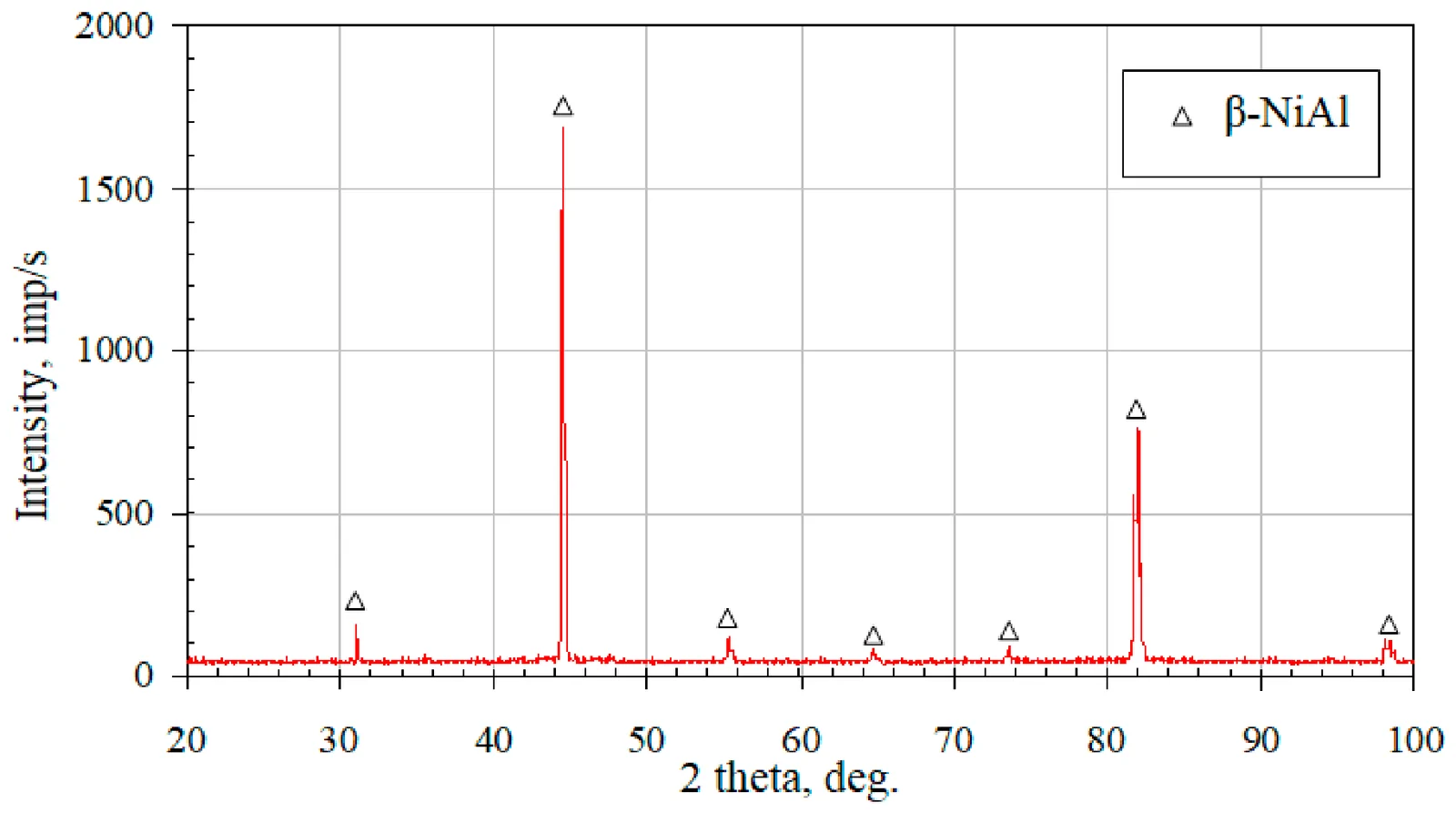
X-ray diffraction pattern of the rhodium- and hafnium-doped aluminide coating on CMSX-4.

Figure 8 shows the X-ray diffraction result for the rhodium- and hafnium-doped aluminide coating specimen. The coating possessed the crystal structure of the β-NiAl phase. No peaks corresponding to precipitates are detected. This is because the volume fraction of precipitates falls below the X-ray diffraction XRD detection limit.

### 3.3. Oxidation Test Results of the Rhodium- and Hafnium-Doped Aluminide Coating on CMSX-4 Substrate

**Figure 9:**
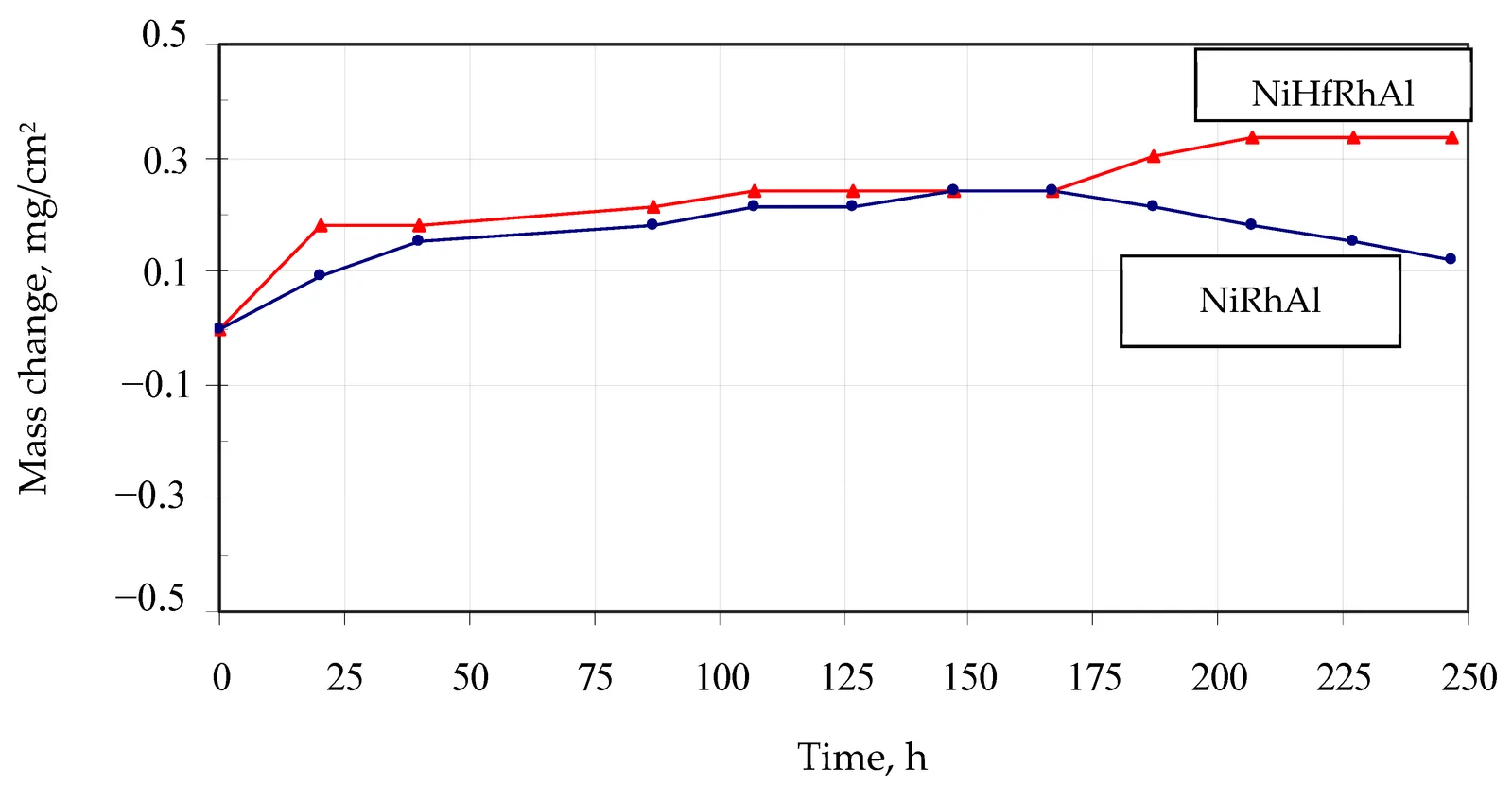
Mass change curves of the doped coatings during the oxidation test.

**Figure 10:**
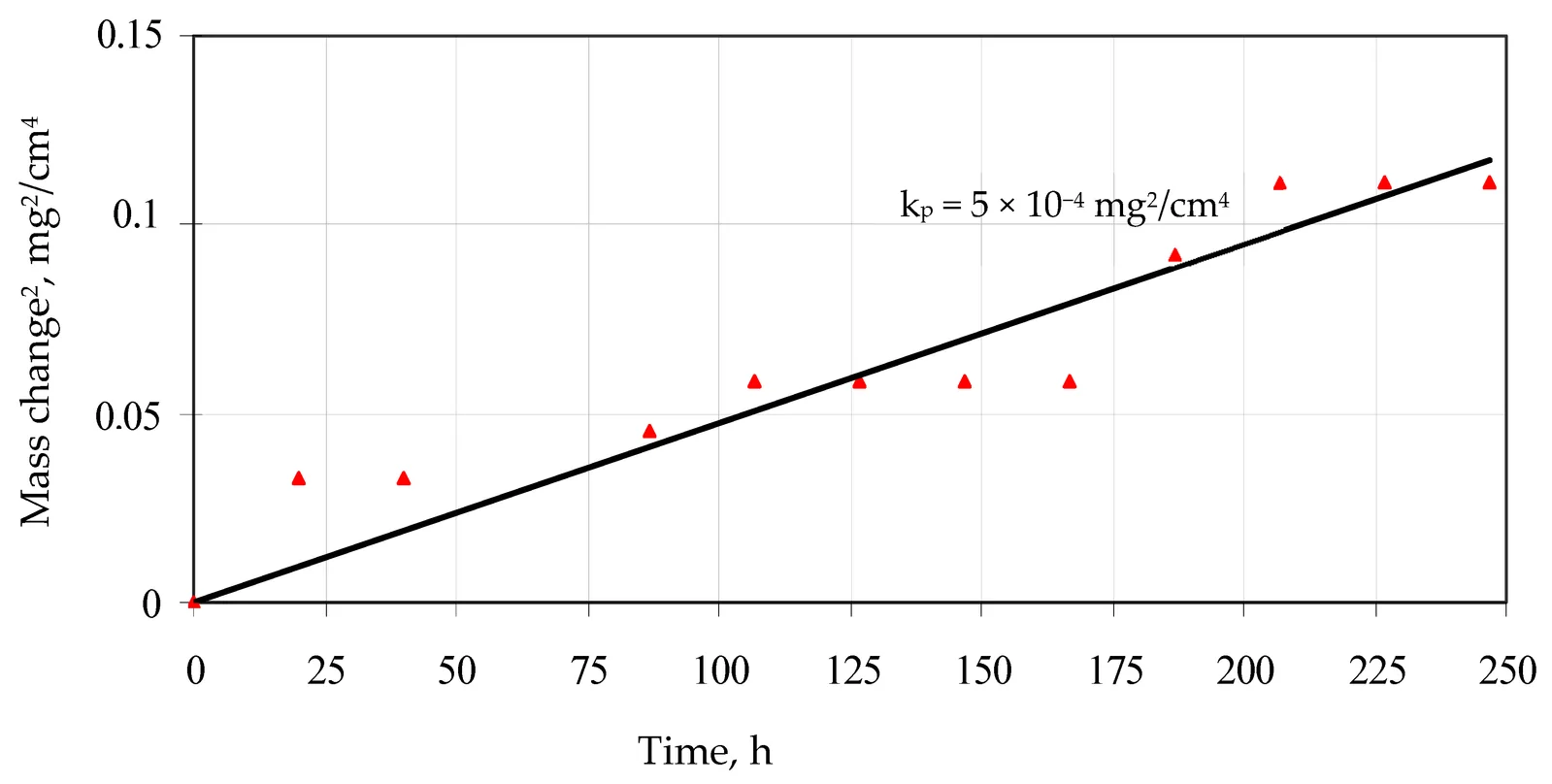
Square of mass change curve of the rhodium- and hafnium-doped aluminide coating on CMSX-4 during the oxidation test.

The curves of the mass change of the doped aluminide coatings during the oxidation experiment at 1100 °C are shown in Figure 9. Rhodium- and hafnium-doped aluminide coatings have shown better resistance to oxidation than those doped only with rhodium. The constant k_p_ value of the oxidation rate of the rhodium- and hafnium-doped aluminide coatings obtained using the slope of the fitted lines was 5 × 10^−4^ mg^2^/cm^4^·h (Figure 10). The fitting range was 0–250 h and the coefficient of determination, R^2^, was 0.8852. The mass of doped coatings increased noticeably during the first hours of oxidation (Figure 9). The rapid mass gain in the early stage of oxidation may be due to the formation of unstable oxides at the surface. Similar kinetics during the early stage of oxidation of NiPtAl coatings on a single-crystal superalloy was observed by Liu et al. [36]. The mass of coatings became stable from 40 to 160 h of oxidation. The stable mass of coatings may be due to the formation of protective oxides at the surface of coatings. Similar oxidation kinetics of PtZrAl coatings on a single-crystal superalloy were observed by Fan et al. [37]. Prolongation of the time from 160 to 240 h led to the mass gain (to 0.33 mg/cm^2^) of the rhodium- and hafnium-doped aluminide coatings and led to mass loss (to 0.12 mg/cm^2^) of the rhodium-doped aluminide coatings. The belt of hafnium-rich precipitates in the rhodium- and hafnium-doped aluminide coatings may block the outer substrate element diffusion and internal aluminum diffusion. Therefore, the rhodium- and hafnium-doped aluminide coating enabled better protection from oxidation than only the rhodium-doped one. Yang et al. observed similar phenomena [38,39,40].

## 4. Discussion

Rhodium- and hafnium-co-doped aluminide coatings were efficiently produced on nickel and CMSX-4 superalloy. Coatings on both kinds of substrates comprised two layers: the additive on the top of the coating and the interdiffusion below the additive one. The chemical composition of the interdiffusion layer on the nickel substrate indicated the presence of the γ’-Ni_3_Al phase doped with hafnium while the chemical composition of the interdiffusion layer on the CMSX-4 substrate indicated the presence of a β-NiAl phase matrix doped with rhodium. The low solubility of titanium, chromium, or rhenium from the substrate resulted in the formation of precipitations of these elements in the rhodium- and hafnium-doped NiAl matrix. The EDS analysis of the additive layer on both kinds of substrates suggested a β-NiAl phase doped with rhodium and hafnium. The presence of the β-NiAl phase was supported by X-ray diffraction results (Figure 8). The β-NiAl phase was also identified in the modified aluminide coatings [25,26].

Small rhodium content of 0.1–0.6 at.% was identified at the additive/interdiffusion layer interface both on the nickel and superalloy. Similar atomic radii of rhodium and nickel cause rhodium to occupy nickel sites in the crystal lattice of the NiAl phase, and β-(Ni,Rh)Al could be formed [41]. No rhodium was revealed in the interdiffusion layer of the coating on the nickel substrate. It corresponded to results for palladium- and rhodium-modified aluminide coatings deposited on the same substrate [42]. Neither rhodium nor hafnium was identified on the surface of the coating on the CMSX-4 substrate.

**Figure 11:**
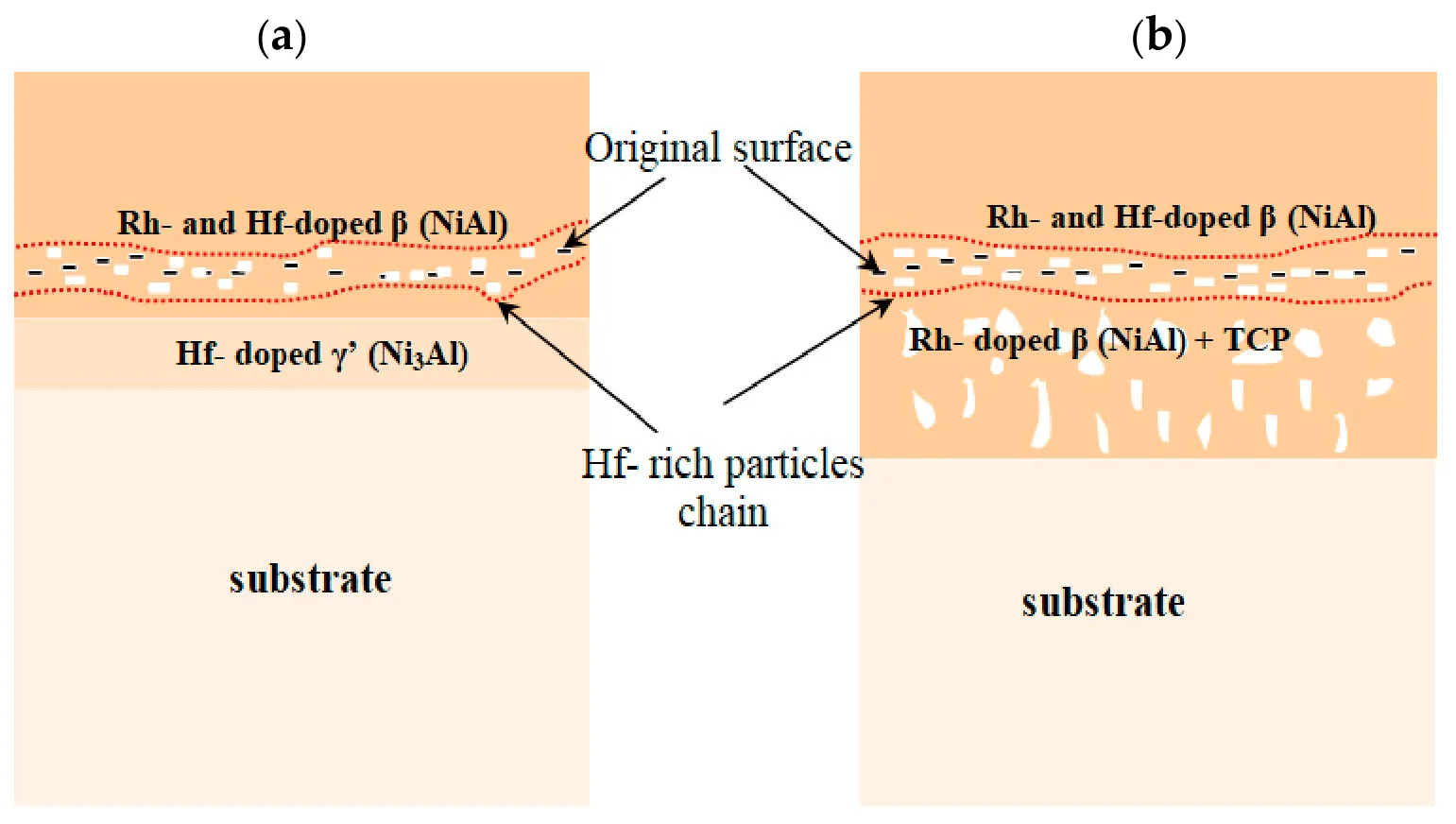
Schematic of the rhodium- and hafnium-doped aluminide coating structure on nickel (**a**) and CMSX-4 (**b**).

Hafnium’s melting point is higher than those of rhodium, nickel, or aluminum. This indicates that hafnium has a higher activation energy of diffusion (*Q*), which is required for vibration of the crystal lattice [25]. A relationship of the diffusion coefficient (*D*) with the activation energy of diffusion (*Q*) and absolute temperature *T* was shown in Equation (4) [25,38].

(4) $$D=D_{0}e^{-\frac{Q}{\mathrm{RT}}}$$

where *D*_0_ is the pre-exponential diffusion coefficient and *R* is the gas constant. A higher value of energy of activation means a lower value of diffusivity. During heating specimens from the ambient temperature to 1040 °C, the rhodium layer could change to a (Ni,Rh) solid solution. Aluminizing at 1040 °C was a low-activity, high-temperature process, as a result of which the rhodium-doped NiAl phase was formed. The growth of the rhodium-doped NiAl coating was mostly determined by the nickel outward diffusion. Further hafnizing with aluminizing led to hafnium-rich precipitate formation. The diffusion of chromium, rhenium, or titanium from the substrate took place at the same time. On the other hand, the low solubility of these elements in the rhodium-doped NiAl phase could inhibit hafnium diffusion. When aluminizing was finished, hafnium-rich precipitates stayed at the original surface of the substrate, resulting in the hafnium-rich particle chain appearance (Figure 11).

The addition of the reactive elements to the aluminide coatings leads to acceleration of the formation of protective oxide on the surface of coatings [43,44,45]. Due to the high affinity for oxygen of reactive elements, oxide particles are formed. Fine oxide particles of reactive elements acted as sites of nucleation of Al_2_O_3_ oxides and could decrease alumina formation inter-nuclei spacing [43]. Thus, less time for the protective oxide layer formation could be required. On the other hand, the reactive element oxide formation could cause the reactive element depletion in the coating. Hafnium-rich particle chains near the additive/interdiffusion layer interface could act as a reactive element reservoir [25]. Since the solubility of hafnium in the β-NiAl phase is very low (<1 at.%), the high temperature of oxidation of doped coatings (1100 °C) could lead to slight dissolution of the hafnium-rich chain [46]. The hafnium-rich small precipitates distributed near the additive/interdiffusion layer interface could ensure the supply of appropriate hafnium content into the rhodium-doped NiAl phase of the coating [25]. It could prolong the life and enhance the oxidation resistance of the doped coating [25]. The oxidation kinetics of Rh-monodoped specimens and the simple undoped aluminide coating on the CMSX-4 superalloy were compared by Zagula-Yavorska [47], whereas oxidation kinetic investigations of simple NiAl, Pt-monodoped NiAl, Hf-monodoped NiAl, and Pt-Hf-co-doped NiAl coatings on the superalloy unequivocally indicate that the Pt-Hf-co-doped NiAl coating had better oxidation resistance than the Pt-monodoped, Hf-monodoped, or simple NiAl coating [48]. Since rhodium is a platinum group element, it can be assumed that the performance improvement arises from genuine Rh-Hf synergy instead of Hf addition alone.

**Figure 12:**
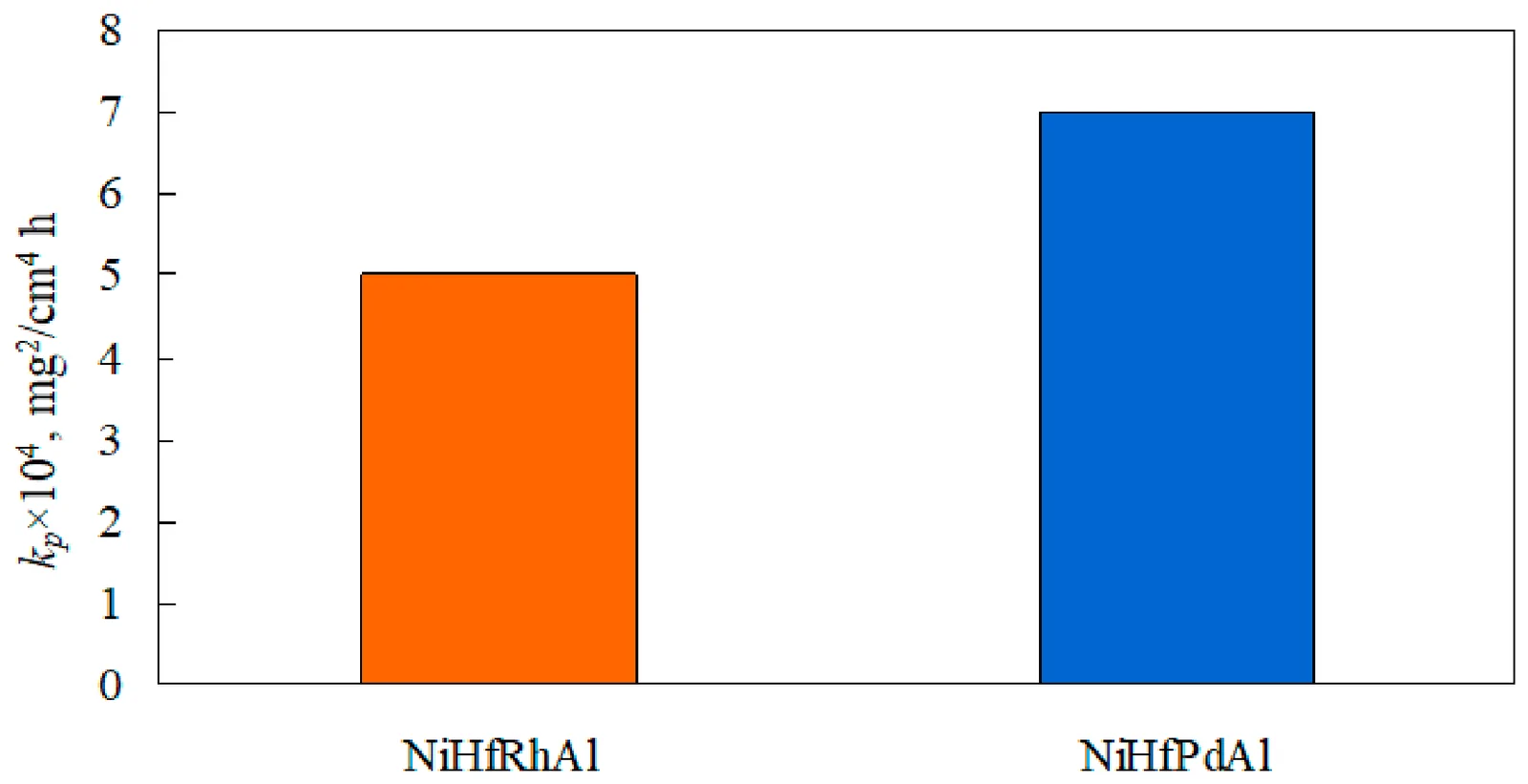
Oxidation rate constant for the rhodium- and hafnium-doped or palladium- and hafnium-doped aluminide coatings.

The mass gain of the rhodium- and hafnium-doped aluminide coatings on the CMSX-4 superalloy is 0.2 mg/cm^2^ after 160 h of oxidation. This is 0.1 mg/cm^2^ less than the mass gain of the palladium- and hafnium-modified aluminide coatings on the same substrate after 160 h of oxidation [39]. This is also 0.16 mg/cm^2^ less than the mass gain of the hafnium-doped (Ni, Pt)Al coating on the single-crystal nickel-based superalloy after 160 h of oxidation [25]. But this is also 0.46 mg/cm^2^ less than the mass gain of the (Ni, Pt)Al coating on the single-crystal nickel-based superalloy after 160 h of oxidation [25]. The k_p_ value comparison of the rhodium- and hafnium-doped aluminide coatings and the palladium- and hafnium-doped ones is based on previously published results [39]. Both kinds of coatings (rhodium- and hafnium-doped aluminide coatings and the palladium- and hafnium-doped ones) are produced and examined in the same laboratory—the Research and Development Laboratory for Aerospace Materials and experimental conditions are comparable. The value of the oxidation rate constant k_p_ of the rhodium- and hafnium-doped aluminide coatings on CMSX-4 is about k_p_ = 5 × 10^−4^ mg^2^/cm^4^·h (Figure 12). This is 2 × 10^−4^ mg^2^/cm^4^·h less than the k_p_ value of the palladium- and hafnium-modified aluminide coatings on the same superalloy [39] (Figure 12).

The obtained results and their analysis indicated that the rhodium and hafnium co-doping slowed the oxidation rate of the aluminide coating. Moreover, rhodium and hafnium co-doping is more efficient than palladium and hafnium co-doping in reducing the oxidation rate of the aluminide coating on a CMSX-4 substrate. The results presented in this study provide an initial assessment of the investigated coating. Further investigations, including longer-term oxidation or thermal cycling tests, coating/substrate adhesion measurements, and a detailed characterization of Hf-enriched particles, would provide additional insight into the oxidation behavior, mechanical integrity, and microstructural evolution of the coating. These investigations are planned as part of future research to further extend the findings obtained in the present study.

## 5. Conclusions

Novel rhodium- and hafnium-co-doped aluminide coatings were prepared through rhodium plating and subsequent hafnizing, combined with aluminizing, using the CVD method. The following conclusions can be formulated based on experimental results:

1. Rhodium- and hafnium-co-doped aluminide coatings on nickel and CMSX-4 substrates are composed of two layers: an additive layer and an interdiffusion layer.
2. The additive layer contained the rhodium- and hafnium-doped β-NiAl phase.
3. The interdiffusion layer on the nickel substrate contained the hafnium-doped γ’-Ni_3_Al phase, whereas that on the CMSX-4 substrate contained the rhodium-doped β-NiAl phase.
4. The hafnium-rich small particles were formed near the additive/interdiffusion layer border.
5. Rhodium and hafnium co-doping was more efficient than only rhodium doping; moreover, it was more efficient than palladium and hafnium co-doping in reducing the oxidation rate of the aluminide coating on the CMSX-4 substrate.

## Figures and Tables

**Table 1:** Nominal chemical composition of single-crystal nickel based superalloy CMSX-4 (wt.%).

Cr	Ni	Co	W	Mo	Ta	Ti	Re	Hf	Al
6.5	Bal.	9	6	0.6	6.5	1	3	0.1	5.6

**Table 2:** Cross-section chemical composition of rhodium- and hafnium-doped aluminide coating on pure nickel (at.%).

Spot	Al	Ni	Rh	Hf
1	36.2 ± 0.2	63.0 ± 0.7	0.5 ± 0.1	0.3 ± 0.2
2	24.3 ± 0.3	75.2 ± 0.8	0.1 ± 0.1	0.4 ± 0.2
3	35.4 ± 0.3	63.4 ± 0.7	0.4 ± 0.1	0.8 ± 0.2
4	-	100	-	-

**Table 3:** Cross-section chemical composition of additive/interdiffusion layer border of rhodium- and hafnium-doped aluminide coating on pure nickel (at.%).

Spot	Al	Ni	Rh	Hf
1	42.7 ± 0.3	55.3 ± 0.7	0.3 ± 0.1	1.7 ± 0.2
2	30.8 ± 0.3	54.2 ± 0.9	0.2 ± 0.1	14.8 ± 0.9
3	35.1 ± 0.3	60.8 ± 0.8	0.5 ± 0.1	3.6 ± 0.6

**Table 4:** Surface chemical composition of rhodium- and hafnium-doped aluminide coating on CMSX-4 substrate (at.%).

Spot	Al	Ni	Cr	Co	Rh	Hf
1	49.9 ± 0.3	45.8 ± 0.3	0.8 ± 0.0	3.5 ± 0.2	-	-
2	43.3 ± 0.3	51.3 ± 0.4	0.9 ± 0.0	4.5 ± 0.2	-	-
3	46.3 ± 0.3	48.5 ± 0.3	0.9 ± 0.0	4.3 ± 0.2	-	-

**Table 5:** Cross-section chemical composition of rhodium- and hafnium-doped aluminide coating on CMSX-4 (at.%).

Spot	Al	Ni	Ti	Cr	Re	Rh	Hf
1	40.8 ± 0.3	57.3 ± 0.6	-	1.1 ± 0.1	0.5 ± 0.0	0.1 ± 0.0	0.2 ± 0.1
2	28.1 ± 0.2	52.5 ± 0.6	0.9 ± 0.1	16.1 ± 0.2	2.1 ± 0.1	0.3 ± 0.1	-

**Table 6:** Cross-section chemical composition of additive/interdiffusion layer border of rhodium- and hafnium-doped aluminide coating on CMSX-4 (at.%).

Spot	Al	Ni	Cr	Re	Rh	Hf
1	70 ± 0.4	26.6 ± 0.6	1.5 ± 0.1	1.1 ± 0.1	0.3 ± 0.1	0.5 ± 0.2
2	36.7 ± 0.9	43.5 ± 1.7	6.5 ± 0.6	6.1 ± 0.5	0.6 ± 0.2	6.6 ± 0.8

## Data Availability

The original contributions presented in this study are included in the article. Further inquiries can be directed to the corresponding author.

## References

[1] Pashapour J., Amidpour M., Torabi F. (2026). Simulation and off-the-shelf integration in an ammonia-fueled hybrid energy system: Merging parallel inverted gas turbines and SOFCs for decentralized power generation. Fuel.

[2] Wadzer Y., Zainuddin A.H., Abdul Halim M.R., Mazlan N.M. (2026). Impact of nanoparticles as an additive to biojet fuel on turbine aviation engine: A performance study. Fuel.

[3] Tüfekci M., Tüfekci E. (2026). A novel analytical beam formulation and its application on composite wind turbine blades. Eur. J. Mech. A/Solids.

[4] Luo C., Yang X., Weng H., Yuan H. (2026). Degradation mechanism of nickel-base single crystal superalloy after long-term aging: Roles of nanomechanical properties and morphology of γ/γ′ phases. J. Mater. Sci. Technol..

[5] Górka J., Lont A., Poloczek T., Żuk M. (2026). The Structure, Microhardness and solid particle erosion wear characteristics of the laser-cladded Inconel 625 coatings reinforced with Cr_3_C_2_ particles on non-alloy steel substrate. Materials.

[6] Chondrakis G., Tzanis A., Georgiou E., Koutsomichalis A. (2025). Root cause analysis of turboprop engine Inconel 713LC turbine blades failure. Eng. Fail. Anal..

[7] Luo C., Weng H., Yang X., Yuan H. (2025). Microstructure-sensitive low-cycle fatigue in a nickel-base single crystal superalloy: Constitutive modeling and life assessment. Int. J. Mech. Sci..

[8] Inceyer A.A., Güven G., Demiralay K., Kazdal Zeytin H., Usta M. (2022). The effects of chemical vapor aluminizing process time and post-processing for nickel aluminide coating on CMSX-4 alloy. J. Mater. Eng. Perform..

[9] Wang W., Li B., Yang B., Li Y. (2025). A thermo-chemo-mechanical fatigue phase-field model of thermal barrier coatings under cyclic high-temperature gas exposure. Int. J. Fatigue.

[10] Manam B.U., Langhof N., Sitzmann C., Schafföner S. (2025). Overview of interfacial fracture toughness testing of ceramic coatings at room and elevated temperatures. Theor. Appl. Fract. Mech..

[11] Guler F., Inceyer A.A., Kol S., Usta M., Aydin H., Keles O. (2025). Improving cyclic oxidation resistance of additively manufactured IN939 superalloy via chemical vapor aluminizing. Adv. Eng. Mater..

[12] Meng G.-H., Li S.-S., Wang Y.-N., Gui P.-P., Zhang M.-Y., Guo K.-Y., Liu M.-J., Yang G.-J. (2025). Unraveling β-NiAl degradation in aluminide coatings: A comparative study of isothermal oxidation and vacuum heat treatment. Corros. Sci..

[13] Zhao Y., Zhou K., Xin X., Zeng X., Guo X., Qiao Y. (2025). Microstructure and oxidation behavior of the aluminized coating on K447A nickel-based superalloy prepared by AlF3-activated pack cementation. Surf. Coat. Technol..

[14] Pint B.A., More K.L., Wright I.G. (2003). Effect of quaternary additions on the oxidation behavior of Hf-doped NiAl. Oxid. Met..

[15] Godlewski K., Godlewska E. (1987). The effect of chromium on the corrosion resistance of aluminide coatings on nickel and nickel-based substrates. Mater. Sci. Eng..

[16] Dai Y.-Z., Zou J.-P., Ning X.-Z., Wei H.-M., Zhan W.-Y., Li F.-Y. (2024). Microstructure evolution and oxidation behavior of silicon-modified aluminide coatings on IN718 superalloy at 1000 °C. J. Cent. South Univ..

[17] Pedrizzetti G., Genova V., Scrinzi E., Bottacchiari R., Conti M., Paglia L., Bartuli C. (2025). Slurry aluminizing mechanisms of nickel-based superalloy and applicability for the manufacturing of platinum-modified aluminide. Coatings.

[18] Wang D., Peng H., Gong S., Guo H. (2014). NiAlHf/Ru: Promising bond coat materials in thermal barrier coatings for advanced single crystal superalloys. Corros. Sci..

[19] Stringer J. (1989). The reactive element effect in high-temperature corrosion. Mater. Sci. Eng. A.

[20] Cochardt A.W., Township W. (1961). High-Temperature Alloys. U.S. Patent.

[21] Guo H., Sun L., Li H., Gong S. (2008). High temperature oxidation behavior of hafnium modified NiAl bond coat in EB-PVD thermal barrier coating system. Thin Solid Films.

[22] Li S., Xu M.M., Zhang C.Y., Niu Y.S., Bao Z.B., Zhu S.L., Wang F.H. (2021). Co-doping effect of Hf and Y on improving cyclic oxidation behavior of (Ni, Pt)Al coating at 1150 °C. Corros. Sci..

[23] Guo H., Li D., Zheng L., Gong S., Xu H. (2014). Effect of co-doping of two reactive elements on alumina scale growth of β-NiAl at 1200 °C. Corros. Sci..

[24] Unocic K.A., Pint B.A. (2013). Oxidation behavior of co-doped NiCrAl alloys in dry and wet air. Surf. Coat. Technol..

[25] Yang Y.F., Jiang C.Y., Yao H.R., Bao Z.B., Zhu S.L., Wang F.H. (2016). Preparation and enhanced oxidation performance of a Hf-doped single-phase Pt-modified aluminide coating. Corros. Sci..

[26] Jiang C., Qian L., Feng M., Liu H., Bao Z., Chen M., Zhu S., Wang F. (2019). Benefits of Zr addition to oxidation resistance of a single-phase (Ni, Pt)Al coating at 1373 K. J. Mater. Sci. Technol..

[27] Sarraf S., Rastegari S., Soltanieh M. (2024). Deposition of a low-activity type cobalt-modified aluminide coating by slurry aluminizing of a pre-Co-electroplated Ni-based superalloy (IN738LC). J. Mater. Res. Technol..

[28] Zagula-Yavorska M. (2025). Effect of Ni-based superalloy on the composition and lifetime of aluminide coatings. Materials.

[29] Xu Z., Dai J., Niu J., He L., Mu R., Wang Z. (2024). Isothermal oxidation and hot corrosion behaviors of diffusion aluminide coatings deposited by chemical vapor deposition. J. Alloys Compd..

[30] Xu Z., Wang Z., Niu J., Dai J., He L., Mu R. (2016). Phase structure, morphology evolution and protective behaviors of chemical vapor deposited (Ni, Pt)Al coatings. J. Alloys Compd..

[31] Liu J., Li Q., Xie Y., Luo J., Zhang C., Wang W., Cao K., Liang X., Yang L., Zhou Y. (2025). Microstructural evolution mechanism of aluminide coating on the inner hollow of superalloy blades during service in a real gas turbine. J. Mater. Sci. Technol..

[32] Li P., Xin Z., Sun F., Jin X., Zhang C. (2025). Microstructure evolution and damage mechanism of DD9 single crystal superalloy-thermal barrier coating system under high temperature oxidation: A comparative study with DD6. Materials.

[33] Kepa T., Bonnet G., Pedrizzetti G., Genova V., Pulci G., Bartuli C., Pedraza F. (2025). Slurry aluminizing of nickel electroless coated nickel-based superalloy. Coatings.

[34] Cui T., Geng S., Niu X., Jia Q., Chen M., Wang F. (2026). Effect of Co-Al co-deposition on high-temperature corrosion behavior of surface-dependent CVD aluminide coatings on Ni-based single-crystal alloys. Surf. Coat. Technol..

[35] Cojocaru M.O., Branzei M., Druga L.N. (2022). Aluminide diffusion coatings on IN 718 by pack cementation. Materials.

[36] Liu H., Xu M.M., Li S., Bao Z.B., Zhu S.L., Wang F.H. (2020). Improving cyclic oxidation resistance of Ni_3_Al-based single crystal superalloy with low-diffusion platinum-modified aluminide coating. J. Mater. Sci. Technol..

[37] Fan Q., Yu H., Wang T.-G., Wu Z., Liu Y. (2018). Preparation and isothermal oxidation behavior of Zr-doped, Pt-modified aluminide coating prepared by a hybrid process. Coatings.

[38] Yang Y.F., Ren P., Bao Z.B., Zhu S.L., Wang F.H., Li W. (2020). Microstructure and cyclic oxidation of a Hf-doped (Ni, Pt)Al coating for single-crystal superalloys. J. Mater. Sci..

[39] Romanowska J., Morgiel J., Kolek Ł., Kwolek P., Zagula-Yavorska M. (2018). Effect of Pd and Hf co-doping of aluminide coatings on pure nickel and CMSX-4 nickel superalloy. Arch. Civ. Mech. Eng..

[40] Lu Y.J., Ren P., Yang Y.F., Wang Q.W. (2026). In-situ formation of a HfO_2_/Al_2_O_3_ ceramic diffusion barrier in (Ni, Pt)Al coatings and its effect on cyclic oxidation behavior. Corros. Sci..

[41] Lamesle P., Steinmetz P. (1995). Growth mechanisms and hot corrosion resistance of palladium modified aluminide coatings on superalloys. Mater. Manuf. Process..

[42] Zagula-Yavorska M. (2023). Synergistic effect of Pd+Rh on the microstructure and oxidation resistance of aluminide. J. Min. Metall. Sect. B Metall..

[43] Hayashi S., Gleeson B. (2009). Early-stage oxidation behavior of Pt-modified γ’-Ni_3_Al-based alloys with and without Hf addition. Oxid. Met..

[44] Rossignol S., Kappenstein C. (2001). Effect of doping elements on the thermal stability of transition alumina. Int. J. Inorg. Mater..

[45] Smialek J.L. (2022). Invited review paper in commemoration of over 50 years of oxidation of metals: Alumina scale adhesion mechanisms: A retrospective assessment. Oxid. Met..

[46] Kim G.Y., He L.M., Meyer J.D., Quintero A., Haynes J.A., Lee W.Y. (2004). Mechanisms of Hf dopant incorporation during the early stage of chemical vapor deposition aluminide coating growth under continuous doping conditions. Metall. Mater. Trans. A Phys. Metall. Mater. Sci..

[47] Zagula-Yavorska M. (2019). Rhodium influence on the microstructure and oxidation behaviour of aluminide coatings deposited on pure nickel and nickel based superalloy. High Temp. Mater. Process..

[48] Wang Y., Smialek J., Suneson M. (2014). Oxidation Behavior of Hf-Modified Aluminide Coatings on Inconel-718 at 1050 °C. J. Coat. Sci. Technol..

